# Impacts of Rumors and Conspiracy Theories Surrounding COVID-19 on Preparedness Programs

**DOI:** 10.1017/dmp.2020.325

**Published:** 2020-09-09

**Authors:** Inayat Ali

**Affiliations:** Department of Social and Cultural Anthropology, University of Vienna, Austria

**Keywords:** conspiracy theories, COVID-19, Pakistan, pandemic, rumors

## Abstract

Being a part of our sociocultural history, stories and narratives help us make sense of our lifeworlds. Stories, rumors, and conspiracy theories offer deep meanings when analyzed in specific contexts, and prominently appear in the face of looming uncertainties, anxieties, and fears. Similarly, many narratives have surrounded the coronavirus disease 2019 (COVID-19) pandemic at the global and local levels as people try to make sense of this invisibly spreading virus and its multidimensional effects. Drawing on the media reports, I show and analyze global-level narratives that reveal geopolitics in play. To present the local level narratives in Pakistan, I build on my long-term ethnographic fieldwork, recent telephone interviews, and content analysis to discuss why these tales emerge and spread. As the pandemic unfolded, local people started drinking “miraculous” tea as a form of prevention, shaving their heads, and/or praying to God to undo His “punishment” and conceptualizing the pandemic as an invented “plot.” With my analyses, I compare the “viral rumors” with the virus and argue that these narratives are social phenomena, carrying multiple meanings that need the thorough attention of social scientists, for example, anthropologists, just as we need experts to study a virus.

Stories and narratives are part of our human sociocultural history. From our early beginnings as foragers, we humans have created and told and retold myths, legends, folktales, and other types of stories, both to help us make sense of our lifeworlds and to encode knowledge and wisdom learned over generations, thereby passing it on. Before the invention of writing, tales were the best sources to preserve and share specific meanings.^1^ Narratives are social phenomena, and many are created and reworked to make sense of and to deal with uncertain and challenging situations. The rapidity of their spread reveals the intensity of the challenge faced. Natural disasters, wars, and contagious outbreaks, epidemics, and pandemics, such as coronavirus disease 2019 (COVID-19), have accumulated multiple narratives. As the number of COVID-19 cases swiftly escalated, so did the rumors and conspiracy theories^2^ that led the World Health Organization (WHO) to coin a new term, “infodemic,” to encapsulate the pandemic of “(mis)information” that rapidly spread by means of social media and word-of-mouth.^3,4^

At the global level, politicians and governments started pointing their fingers at each other. For instance, the United States and China made competing narratives to blame each other that they are the creators of this virus. At local levels, people brainstorm probable causes of COVID-19 and circulate advice about preventions and “cures,” and at all levels, rumors have spread about the potential agents behind this virus. When these narratives take the form of rumors and conspiracy theories, it is indispensable to document them for a thorough understanding of how people make sense of a looming threat, and most crucial to question the underlying reasons for these speculations and their impacts on the preparedness programs: Why do these narratives start and what do they reveal? How do people culturally construct, generate meanings, and negotiate the outbreaks of disease, particularly COVID-19? What implications will these have on programs of preparedness at a local, national, and global level?

Precisely, studying viral rumors and conspiracy theories is as crucial as researching the virus to lucidly comprehend the content, sources, modes of spread, and impacts of these narratives.

## METHODS

I draw on my ethnographic fieldwork on cultural understandings of health and illness, especially negotiations of infectious diseases, including vaccination in Pakistan, which I commenced in 2008 and have continued to date. In addition, while living in Austria during the COVID-19 pandemic that broke out in my country in February 2020, I have been having phone conversations with my family, friends, and acquaintances in Pakistan to thoroughly understand how COVID-19 is perceived, negotiated, and dealt with at village, provincial, and national levels. These data are based on convenience sampling. The data I analyze herein also come from the content analysis of mainstream social media reports that I began to undertake in January 2020. For providing a historical outline of rumors and conspiracy theories, I borrow from the relevant and extensive body of literature, largely from psychology and anthropology. The study is part of a project by the National Bioethics Committee Pakistan (reference No.4-87/NBC-471-COVID-19-09/20/).

### Global Conspiracy Theories Around COVID-19

Globally, conspiracy theories are revealing geopolitics. A conspiracy theory emerged that, although there is no evidence that the United States has bioengineered the virus, the country has deep interests in affecting China’s economy.^2^ Likewise, China and Russia are pointing fingers at the United States, for example, Chinese officials claimed that the US Army had introduced the virus to China.^6^ The Venezuelan President also argued that the United States used the virus as a “bioweapon” for targeting China.^7^ Turkmenistan and Tajikistan asked their citizens to continue working after calling the virus and its treatment “bogus.”^7^

In contrast, Matteo Salvini, the leader of Italy’s anti-migrant League Party, contended that the outbreak of the virus is China’s doing, insisting that the Chinese have deliberately cultivated a “lung supervirus” from “bats and rats.”^7^ Likewise, the United States blamed China for the origin and early transmission of the virus: US Senator Tom Cotton suspects that perhaps China produced the virus in its weapons lab^7^—a rumor that went so far that it is currently being addressed on US National Public Radio. President Trump started calling the virus a “foreign virus” and “the China Virus,”^5^ warned the WHO against being so “China-centric” and he is cutting funding to the organization, although the WHO firmly rejected that criticism.^8^

Furthermore, on social media, especially on Facebook and Instagram, people shared speculations that Bill Gates, on behalf of Big Pharma, is behind the emergence of COVID-19. People refer to his TED-talk of 2015, in which he argued that the next viral outbreak might prove more deadly than war.^9^ Clearly, predicting the occurrence of something can lead people to believe that you created that something, perhaps just to prove yourself right.

These news reports, social media videos, and stories have accumulated a plethora of comments that discuss, argue, and counter-argue these stories. These comments demonstrate how people are negotiating the pandemic by finding a scapegoat. People often need somewhere to go with their anger and confusion; blaming and shaming serve that purpose and make the people doing it feel powerful.

### Rumors and Conspiracy Theories in Pakistan’s Villages and Towns

In this section, I draw on various competing narratives with unknown and untraceable sources that have emerged in Pakistan, especially in villages. These narratives are not exceptional, as rumors and conspiracy theories about infectious diseases’ outbreaks have long been prevailing there. Various rumors surround vaccination programs—these are a means of “depopulation” by means of the sterilization of Muslim women and that hidden stakeholders are behind these campaigns.^10^ About COVID-19, comparable stories have surfaced to trace a “hidden” agent: the pandemic is “bioengineered” either by the United States or by Big Pharma. Speculations also contain home remedies, such as drinking garlic water (which might actually help), or “blowing hot air from a hairdryer through your nostrils”; the country’s health ministry suggests keeping your throat moist.^11^ Many people are following these (relatively harmless) suggestions.

Particularly, I describe and analyze 6 specific narratives currently circulating in Pakistan: (a) In a small village of Sindh Province when the pandemic was still unfolding, a rumor spread that the government sets the infected people ablaze to eliminate and contain the virus.^2^ (b) A comparable rumor surfaced in the small towns of Azad Jammu and Kashmir (AJK): The government shoots the infected persons to contain the virus.

Moreover, extraordinary narratives emerged as the pandemic grew. (c) In Punjab province’s 1 town, the narrative started that COVID-19 is the punishment of God, which is a result of the opening of cinemas in Saudi Arabia and general disbelief in God in the Global North, where everything is “too open,” especially romance. God has shown us how powerful He is in that science is unable to deal with the pandemic. Because Pakistanis believe in and worship God, their number of cases is far less than in the “Godforsaken” countries of the United States and Europe.

(d) Yet a different rumor surfaced in Sindh and Punjab provinces that questioned the existence of coronavirus: How is it possible that one gets infected by means of shaking hands or standing close to someone? The rumor continues that the government is only imposing lockdowns to receive global attention for potential foreign aid and that there is no danger.

(e) A widespread rumor broke out in Sindh province that shaving one’s head protects against the virus. Following this rumor, innumerable men (the shaving of a woman’s head is considered shameful) immediately shaved their heads considering an economically affordable preventive measure that costs only US5-10 cents. In 1 village of Sindh, over 50 men shaved their heads.

(f) The last rumor is concerning a miraculous birth—of an infant who started talking after his birth—that emerged in the northern Sindh, which rapidly spread in many districts. The following is the rumor: “I will not survive. I am here to tell you something important about the current coronavirus that the disease is deadly. I will die at noon and will bring the coronavirus with me,” said the child. It could kill everyone if a recommended measure was not taken. The measure was brewing green tea, and every person should drink 5 sips. The one who would drink these 5 sips would survive; the rest would die. “As long as my heart beats, I ask you to please drink tea.” After conveying this message, the child died.

Showing their concern, local people promptly made efforts to reach their families and friends by means of mobile calls and messages and paying in-person visits.

### Implications of These Narratives on the Preparedness Programs

The global level narratives would significantly affect the preparedness programs, not only related to COVID-19 but also other similar futuristic programs. On the one hand, linking the virus with the geopolitics would affect the programs of preparedness in terms of (in)effective political policies and lack of economic resources not only at a country level but at the global level. On the other hand, these narratives that the virus is a conspiracy of any “super-power” would significantly affect the local level perceptions and practices toward the virus. These competing narratives would testify to not falsify the similar local level narratives—as mentioned earlier, that some people believe that there is no virus.

The local level narratives have adverse and positive effects on behaviors that may considerably affect the preparedness programs. The first 2 (a and b) rumors—burning or shooting infected people—can compel local people to avoid getting the COVID-19 test. Yet both may lead people to adopt preventive measures not to contract the virus due to that fear of death by the government. As these rumors compelled family members, particularly (older) parents, to become highly concerned about the mobility of their younger generation and to ensure that they all stayed at home. In this specific case, we can see that rumors can have positive, instead of solely adverse effects.

The rumor (c), that the virus is “an act of God,” illustrates religious beliefs and gives people hope that the COVID-19 may be successfully dealt with through worshiping God. The rumor (d), that there is no coronavirus, reveals the mistrustful relationship between people and the government. Both rumors can be seen against the historical backdrop of the country and region: colonization, poverty, and aid dependency.

The rumor (d), “shaving one’s head,” is relatively harmless and seems to reveal people’s desire for disease prevention by any means suggested, as does the rumor (e) “drinking 5 sips of green tea.” This rumor has some basis in fact because studies have shown the positive effects of black or green tea (as well as garlic) on the human immune system, although solely 5 sips are unlikely to help much. Moreover, the number “5” has a special meaning within the local belief system. Many people across the country, specifically those who practice Sufism and Shia-Islam, regard numeral 5 as lucky and sacred. They link this number to *Panjtan Pak* (lit. “the Holy Five”), who include Prophet Muhammad, his cousin Ali, daughter Fatima, and their sons Hassan, and Hussain. People believe in their supernatural powers to be advocates to God for seeking help and blessings in normal, and especially during extraordinary, events. In this way, “drinking 5 sips of tea” has physical and symbolic powers—the properties of tea and the symbolic power of 5—both can exert positive impacts on people’s physical and mental bodies. Precisely, both rumors are a good example of how a society can produce an easily accessible “cure” in the absence of an efficient health-care system.

All and all, these local level narratives might have severe implications for the preparedness programs: either people ignore the recommended measures, that is, physical distancing, avoiding handshakes and hugs, or not organizing public gatherings. One might assume that the last rumor about belief in the “Holy Five” would exert critical effects on the recommended measures as local people would easily participate in the religious activities that generally are performed during the first month of the Islamic calendar by the practitioners of Shia-Islam.

### Why Do Rumors and Conspiracy Theories Emerge, and How Do They Travel?

I argue that rumors and conspiracy theories constitute “normal social phenomena.”^12^

While devoting considerable attention to document rumors and conspiracy theories, it is crucial to ask a simple question: Why do these stories emerge and receive wide attention? The simple answer is because, as previously noted, these narratives provide meanings and empower people to feel some sense of control in the face of frightening situations. In addition, these narratives are mysterious and captivating^13^ and can bring excitement to people’s lives. More complex answers involve specific motives that lie behind people’s desire to believe, which include epistemic factors that are required to sufficiently understand one’s environment; the existential need to feel safe and in control of one’s environment; and the social need to maintain a positive image of oneself and one’s in-group.^14^

Before the presence of mass media and social media, word-of-mouth was the primary source of rumor transmission. This historical source of circulation still prevails in those societies where access to media is low.^10^

These narratives may make people feel special—that they possess some essential and rare information that enhances their self-esteem.^15^ Some people deliberately craft specific conspiracy theories, and others produce counter-narratives; hence, in a way, both equally participate in their spread,^14^ as to generate a counter-narrative to a given conspiracy theory is to give attention to and to further spread that theory. Rumors index ambiguities,^16^ convey coalescences of meaning, and reveal interconnections and interplays between local and global contexts. The speculations grow successfully in specific contexts: social, cultural, economic, and political.^14^ Their circulation continues in waves of what I term “the provision of meaning.” Rumors tend to occur in multiple versions and can constitute what is referred to as “global mass hysteria.”^17^ They describe the relationships among (geo)political and economic low-power and high-power groups. The rumors can explicate and indicate the politics of inequality.^16^ Therefore, their meanings must be deciphered within their historical, socio-cultural, economic, and (geo)political contexts to be fully understood.^18^

Rumors can illuminate a lack of power and can allow the less powerful to express their fears. When local people see themselves as powerless against the dominant institutions, for example, the government or international nongovernmental organizations (INGOs), they spin rumors.^19^ To meet an important objective, such narratives assist people to “create an alternative public sphere and maneuver to speak in oppositional voices.”^20^ Conversely, rumors can be created and spread by people who enjoy a great deal of power and wish to use that power to promote their agendas.

In addition to giving power to the powerless and more power to those already enjoying it, rumors can help to negotiate a given phenomenon.^21^ Although rumors can be true or false, or half-truth-and-half-lie,^15^ they are always a form of communication, and they can convey hidden and vital meanings, reflecting beliefs and perceptions regarding the workings of the world, and collective explanations and illustrations about complex circumstances.^22^

Rumors often have their roots in social institutions.^23^ They can function to mobilize crowds and to unite rebellious masses.^24,25^ Rumors differ from other types of communication because of their enunciative and performative functions.^26^ Due to their flexibility, rumors work as a practical mode to reflect upon hard economic and political situations.^16^ In rumors, words transform from a medium of communication to an apparatus of force.^27^ Rumors can disappear and reappear during similar circumstances but with prominent modifications—additions and subtractions—such as in Indonesia.^28^ For a rumor to have a powerful effect, it does not matter whether it is true or false. A rumor containing unofficial information can be false, and so can “official” information.^12^ For their believers, rumor always counts as accurate and meaningful. Perhaps their credibility is unrelated to the rumors themselves but rather to the credibility of the tellers. The authentication of anything can be challenged.

### Comparing “Viral” Rumors and Conspiracy With Viruses

Although the coronavirus and the rumors and conspiracy theories circulating in response to it appear to lie at 2 different poles, they have significant similarities. As viruses are a reality, so are these competing narratives.

COVID-19 is a viral infection. Viruses always have an origin: they emerge, spread, and sometimes cause a major outbreak. Such is the case with COVID-19, which appears to have originated from the Huanan seafood wholesale market in Wuhan, China, then was rapidly transmitted from person to person and then countries to continents.^2,4^ It has affected people socially, culturally, economically, and in terms of their health—physical, emotional, psychological. It has challenged our leaders and global stakeholders, such as the WHO, to effectively deal with it.

For studying the behaviors and patterns of viruses, we have experts, such as virologists, microbiologists, and epidemiologists, who appropriately investigate them. They explore where exactly a virus broke out, why and how it emerged as well as spread so rapidly, what are its modes of transmission and patterns of spread. Under what circumstances can it breed further?

Correspondingly, rumors and conspiracy theories also have specificities that need to be thoroughly studied, adequately understood, and amply illustrated. Rumors and conspiracy theories also emerge in specific situations and diverse places and travel at a pace that is almost as rapid as the virus itself. Like the outbreak of this disease, these narratives affect multiple aspects of life and have their own set of researchers—specialists such as social scientists, especially anthropologists—who can systematically study the patterns, modes, and effects of these narratives. Unlike a viral outbreak, it is usually not possible to trace the origins of these narratives. Yet, it is entirely possible to locate in which area they started, under what circumstances they broke out, what meanings they are pregnant with, and what are their short-term and long-term consequences.

Another critical commonality shared by viruses and rumors is the near-impossibility of stopping their spread or of completely eradicating them, as indicated in the saying, “The rumor went viral.” Various viruses (eg, measles) re-emerge from time to time and place to place, and new viruses emerge (eg, the novel coronavirus causing COVID-19). Viruses can be classified into categories in terms of their similarities, such as the severe acute respiratory syndrome-coronavirus (SARS-COV) family of viruses, of which the coronavirus is a part. Similarly, rumors can be categorized, such as those related to medical or economic arenas. Also, they affect each other—viruses generate rumors and conspiracy theories, and those narratives can affect how people deal with those viruses. During pandemics, rumors and conspiracy theories can adversely, or positively, affect preparedness programs.

Two additional commonalities between viruses and rumors are that both are highly contagious and affect “at-risk” populations. A virus severely affects those who are “weak” in terms of their immune systems, and that “weakness” often stems from their socio-cultural, economic, and political situations. That same population is especially vulnerable to the effects of rumors and conspiracy theories. These factors are critically interdependent and intricately interrelated.

Just as microbiologists and epidemiologists can help to deeply understand a virus to contain and deal with it, so social scientists can help to meticulously understand, contain, and deal with viral rumors, especially anthropologists who are highly equipped, well-positioned, and appropriately skilled to study micro-level phenomena (eg, rumors) and link them to macro-level structures (eg, economics and politics).

### Limitations of the Study

This study also has certain limitations. Because it draws on convenience sampling, the results cannot be generalized to the rest of the country’s population, although the study provokes interesting debates to compare rumors and viruses. All the data is either obtained from the media content or the telephone interviews, which may again affect the results of this study.

## CONCLUSIONS

Studying viral rumors and conspiracy theories is as crucial as researching the virus to lucidly comprehend the content, sources, modes of spread, and impacts of these narratives. Focus can be on factors that lie underneath these stories: why do they spread, in which circumstances and in which parts of the world, and who are their beneficiaries? In this article, I have sought to provide answers to these questions.

As an inadequate understanding of the coronavirus hinders effectively dealing with it at the medical level, so does lack of understanding of the rumors and conspiracy theories surrounding it hinder dealing with this pandemic, the preparedness programs at societal levels. These narratives beg the same paramount attention as making the scientific inquiries of the virus.

It is indispensable to understand that constructing stories—weaving meanings and deciphering them—is a longstanding human social phenomenon. We cannot survive without narratives. Functioning as building blocks of society and as means of enculturation, these narratives help us to encode crucial information that is necessary for biological, sociocultural, emotional, psychological, economic, and—the most important—political survival. It is my hope that my presentation and analysis of the rumors and conspiracy theories surrounding COVID-19 will empower others to understand their sources and effects, both negative and positive, and to find better and more effective means of spreading truths that can also be encoded in narratives—our most popular and longstanding form of information dissemination.
